# Digital Inequalities in the Use of Self-Tracking Diet and Fitness Apps: Interview Study on the Influence of Social, Economic, and Cultural Factors

**DOI:** 10.2196/mhealth.9189

**Published:** 2018-04-20

**Authors:** Faustine Régnier, Louis Chauvel

**Affiliations:** ^1^ Institut National de la Recherche Agronomique Alimentation et Sciences Sociales Unité de Recherche 1303 University of Paris Saclay Ivry sur Seine Cedex France; ^2^ Institute for Research on Socio-Economic Inequality University of Luxembourg Esch-sur-Alzette Luxembourg

**Keywords:** diet, digital divide, fitness trackers, France, healthy diet, physical activity, social networking, social participation, weight loss

## Abstract

**Background:**

Digital devices are driving economic and social transformations, but assessing the uses, perceptions, and impact of these new technologies on diet and physical activity remains a major societal challenge.

**Objective:**

We aimed to determine under which social, economic, and cultural conditions individuals in France were more likely to be actively invested in the use of self-tracking diet and fitness apps for better health behaviors.

**Methods:**

Existing users of 3 diet and fitness self-tracking apps (Weight Watchers, MyFitnessPal, and sport apps) were recruited from 3 regions of France. We interviewed 79 individuals (Weight Watchers, n=37; MyFitnessPal, n=20; sport apps, n=22). In-depth semistructured interviews were conducted with each participant, using open-ended questions about their use of diet and fitness apps. A triangulation of methods (content, textual, and quantitative analyses) was performed.

**Results:**

We found 3 clusters of interviewees who differed by social background and curative goal linked to use under constraint versus preventive goal linked to chosen use, and intensity of their self-quantification efforts and participation in social networks. Interviewees used the apps for a diversity of uses, including measurement, tracking, quantification, and participation in digital communities. A digital divide was highlighted, comprising a major social gap. Social conditions for appropriation of self-tracking devices included sociodemographic factors, life course stages, and cross-cutting factors of heterogeneity.

**Conclusions:**

Individuals from affluent or intermediate social milieus were most likely to use the apps and to participate in the associated online social networks. These interviewees also demonstrated a preventive approach to a healthy lifestyle. Individuals from lower milieus were more reluctant to use digital devices relating to diet and physical activity or to participate in self-quantification. The results of the study have major implications for public health: the digital self-quantification device is intrinsically less important than the way the individual uses it, in terms of adoption of successful health behaviors.

## Introduction

### A Digital Society

We have entered a digital society. Digital technologies are driving economic and social transformations, particularly in the areas of diet and fitness. Self-tracking devices are becoming increasingly prevalent and are changing how individuals monitor their health to a more preventive approach, allowing the general public wide access to data related to health and personalized recommendations [1]. Personal physiological self-tracking has therefore become a very commonplace activity [2].

An increasing number of health-related apps are available for download, and the majority relate to diet, weight, and physical exercise [3]. In the United States, 60% of US adults track their weight, diet, and physical activity on a daily basis [4]. In France, within the context of increasing diet and health inequalities and a reduced habit of quantification, 24% of the population in 2016 used a digital device for their health [5].

Research on diet- and health-related digital devices has produced contrasting results, some studies demonstrating the positive effects of digital devices on dietary behavior change, weight loss, or physical activity [6-9] and others highlighting the limits of such digital devices [10]. Moreover, research has not yet precisely determined the social circumstances under which such apps are beneficial, particularly as active engagement of users is a major issue in digital health [11].

More recently, studies have been conducted among the general public to determine how social differences influence the use of digital self-tracking devices [12]. Ng et al [13], for instance, reported that physical activity trackers are used more by adolescents from affluent milieus. Sociological research has taken a particular interest in how digital devices form a new mode of self-governance and self-measurement. Lupton’s central analysis was of digital devices in the context of the health imperative of contemporary societies [14]. The findings of this study underscored the risks of standardizing practices and warned against the surveillance and self-control aspects of digital devices, which form the basis of new efforts to normalize and standardize behavior [15].

### Daily Uses of Self-Tracking Apps

Regardless, research that provides field data on how these tools are used by individuals in their everyday lives, particularly in the area of dietary habits, is limited. Yet, the question of use is decisive: effectiveness of digital devices may depend less on the tool and more on how the individual uses it [16]. Indeed, digital devices offer several functionalities: measuring (food intake or physical activity), recording (keeping a written record), quantifying (expressing content numerically in digital format rather than in words [2]), and participating in social networks (sharing, commenting on, and comparing data).

The goal of this paper was to analyze the uses and perceptions of digital diet and fitness devices in daily life and their links to social status. The paper will address the following questions: Why do some individuals turn to self-tracking tools at a given moment in their lives? What functionalities do users favor and how do they make use of the digital communities? Finally, what economic, social, and cultural conditions lead individuals to use digital devices actively to attain better health behaviors?

## Methods

To understand the diversity of practices and perceptions and the individual experiences of the participants, our study was based on a qualitative survey. We used the 32-item Consolidated Criteria for Reporting Qualitative Research checklist [17].

### Study Design and Participants

We conducted in-depth, individual, semistructured interviews with 79 individuals. Participants were asked open-ended questions about their use of digital devices in daily life: reasons for choosing the particular device, frequency and circumstances of use, functionalities used, information taken into account when using the device, and effect of use on dietary or sporting habits.

The inclusion criterion was use of at least one of the following self-tracking tools: sport apps (n=22), MyFitnessPal (n=20), or Weight Watchers app (n=37). The 22 individuals in the sport apps group were characterized by the use of common self-tracking tools accessible via cell phone apps, bracelets or watches with accelerometers, and instruments equipped with global positioning system. MyFitnessPal, one of the most popular diet apps [18], is an online calorie counter based on a food diary model. In addition to recording and quantify food intakes, the app determines a recommended daily calorie intake based on the user’s profile (height, weight, gender, daily activity level, and personal objectives in terms of weight).

Both sport devices and MyFitnessPal enable users to store and share data and also to facilitate community discussions, either on the app’s website or via websites for athletes, such as Strava.

Members of Weight Watchers France, a private company that has developed weight loss programs, follow a dietary plan and attend weekly support group meetings [19]. They are offered a mobile app that calculates and records meals via a points system and scans products. They have access to the Weight Watchers website (recipes and discussion forums). Furthermore, members have the option to choose between a digital or paper Weight Watchers program, enabling us to evaluate reasons for choosing a digital app.

The sample was chosen to compare the social status of participants, based on the declared profession, according to the National Institute of Statistics and Economic Studies classification. To do this, interviews were conducted in Paris (n=35), which provided access to high- and middle-income earners residing in urban areas; in and around La Rochelle, Western France, which provided access to middle-income earners (n=13); and, in Eastern France around Thionville, in small towns impacted by the de-industrialization crisis (n=13), versus a wealthy city (n=13)

Participants were recruited from sports clubs or while participating in sporting activities (face-to-face approach), via snowball sampling on the MyFitnessPal social network (initial participants were recruited via the forum and the messaging systems), and via our participation in Weight Watchers meetings.

They were interviewed for 1-2 hours, most often in their own home. We conducted 5 interviews by phone because of the wide geographic distribution of MyFitnessPal participants (ie, more than 3 hours away from Paris, Thionville, or La Rochelle).

All interviews were recorded, transcribed, and anonymized. The transcription was realized by a team of transcription consultants, trained for homogeneity in the processing. The researcher who conducted the field survey was helped by 1 sociologist consultant trained in the processing of semistructured interviews. She was warmly welcomed by participants who were excited to relate their perspective on digital devices. The researcher’s lack of knowledge in the field of self-tracking was a positive characteristic, encouraging participants to adopt the position of expert in the field. Moreover, the researcher’s nonjudgmental attitude toward losing weight enabled participants with weight issues to feel confident.

Interviews were completed with ethnographic observations regarding the uses of digital apps (participants explained their personal health or fitness data, showed us how they had evolved through time, and explained in practical terms how their app worked). Qualitative data were also collected from field notes. The size of the sample made it possible to achieve sufficient saturation, that is, each new participant in each group did not bring any new substantial, relevant knowledge to the survey, and provide enough internal variation to draw solid conclusions regarding differences between practices and perceptions in relation to the social status of individuals.

### Ethics

The goals of the research were explained to the interviewees and their consent was obtained for recording. The Weight Watchers field work was carried out with the approval of the management of Weight Watchers France. All the interviews were strictly anonymized, and interviewees were given fictitious names. The interviewees were thanked with a gift card (€15).

### Quantitative Analysis of Practices

Analysis was based on a triangulation of methods [20]: content, textual, and quantitative analyses. We considered 25 different practices (see Table 1) deriving from the corpus of 79 interviews generating 759,240 words. This corpus was first submitted for content analysis: the data were coded, following the themes of the interview guide and including new themes derived from the data collection (eg, a search for precision vs trend). The content analysis leads to the identification of 6 main themes (functionalities used, uses, engagement in the digital network, relation to publishing, familiarity with information and communication technology (ICT), and reasons for use; sample quotes are given for each theme in Table 1).

The content analysis was double-checked with Hyperbase, based on the specificity tables drawn up for each interview by Hyperbase, using Z-scores which measured the over- or under-representativeness of a word in an interview with respect to the corpus as a whole.

These 6 identified themes lead to the characterization of 25 different practices, measured as dichotomous variables (Table 1). A principal component analysis was carried out using Stata software on the 25 active variables determined by practice. As a robustness check, we conducted a multiple correspondence analysis that produced very similar results. The first 2 axes, accounting in total for 26% of the total variance of the sample, generated a correlation circle [21], representing the 25 active variables (Multimedia Appendix 1); the illustrative (sociodemographic) variables are represented on the principal plan (Multimedia Appendix 1).

Beyond the 2 first dimensions of the factor analysis, we retained 4 additional axes on the basis of differences in variance (other choices did not substantially affect results). These first 6 scoring axes, which accounted for 54% of the variance, were used as active variables of a hierarchical ascending classification (Ward method, squared Euclidean distance) designed to provide a coherent grouping of the population on the basis of their practices. The clusters are represented on the principal plan of the principal component analysis, and the significance of the correlation between cluster group and the 25 active variables (Phi coefficient) is presented in Multimedia Appendix 1.

### Sociodemographic Characteristics

The sample included 60 women, which was attributable to the high proportion of women enrolled in Weight Watchers and using MyFitnessPal. The majority of sport app users were men. Interviewees were aged between 23 and 70 years, with a mean age of 43 years. Most of them were employed in intermediate professions, such as technicians or nurses (31/79, 39%), or were clerical workers (21/79, 27%). Interviewees also had diverse social profiles, in terms of occupational status, associated with their respective self-tracking device. Sport app users tended to belong to high-income milieus, users of MyFitnessPal to the intermediate categories, and Weight Watchers members to intermediate- to low-income milieus (Table 2). Among sport app users, we found that 8 participants used watches (Garmin, Suunto), 5 used mobile phone apps (Runkeeper, Runtastic), 2 used Fitbit wristbands, 6 used 2 devices (mostly a watch plus a smartphone app), and 1 used connected running shoes. We found that 39 individuals used several digital apps at the same time: one to monitor food and another to monitor physical activity. More than half of the Weight Watchers members (20/37, 54%) and 70% (14/20) of MyFitnessPal users used a physical activity tracker. Among sport app users, 23% (5/22) used or had previously used MyFitnessPal.

**Table 1:** Twenty-five variables determined by different uses, categorized by theme and examples of quotes. ICT: information and communication technology; WW: Weight Watchers.

Theme and quote		Variable	Abbreviation	
**Functionalities**				
	“I don’t use MyFitnessPal to count calories.”	Quantification	Quantific	
	“The top functionality, it’s the scan.”	Scan	Scan	
	“The recipe’s calculator is really great!”	Recipes	Recipes	
	“When we cook, we publish our recipes (...) mainly on Facebook (...) because the internet community...chatting with people I don’t know, well...no.”	Facebook_WW	Facebook	
**Uses**				
	“It’s more for myself, to improve over previous years.”	Improvement_self	Improv_self	
	“You know what you have to beat [the record of the guy on the segment] so it's pretty fun.”	Challenge	Comparison	
	“I wanted to know if it (commuting to work via bicycle) took me 32 minutes, which days it’d take 31, when it’d take 28.”	Precision	Precision	
	“It gave me a general idea of whether it was a good or not-so-good session.”	Trend	Trend	
	“It’s good to be able to compare from one time to another one.”	Correlate_data	Correl_data	
**Digital network**				
	“I was totally focused on the community.”	Community_yes	Com._yes	
	“No, I don’t use the forum, I’m not active.”	Community_no	Com._no	
	“At the beginning, I was on the forum, but I only read (the posts).”	Passive_engagement in digital social network	Passiv_eng	
	“So, I did publish much.”	Active_engagement in digital social network	Active_eng	
	“The community is awesome.”	Support	Support	
**Relation to publishing**				
	“When I was a child, I trained in a club, and yes, we used to fill out our cycle rides, with the number of kilometers, on a calendar.”	Anteriority of written record keeping	Anteriority	
	“I have my little... my little Weight Watchers booklet (...) I wrote on my little paper notebook.”	PaperWW	PaperWW	
	“I mean, you never know with Internet or apps, so I prefer being careful.”	Fear	Fear	
**Familiarity with information and communication technology**				
	“I work in an office...I’m an accountant so we’re used to working with Excel, with Word, with several types of software.”	ICT_familiarity without self-quantification	ICT_fam	
	“But at home, I don’t use a computer...nor a laptop, except for online games, in the evenings to relax.”	Digital_Entertainment without self-quantification	E_entertain	
**Reasons for use**				
	“I consider my body as an unfinished piece of art (...) that you constantly try to improve, to shape, to sculpt.”	Esthetic	Esthetic	
	“I have never had health issues because of my weight. It’s for prevention.”	Preventive	Preventive	
	“I suffer from diabetes (...) I had to give my doctor my food diary (...) With the app (MyFitnessPal) I took screenshots and I printed them.”	Chronic illness_management	Chronic	
	“I wanted to lose weight,” “I really wanted to lose weight.”	Curative_weight	Curative	
	“The marathon was my target.”	Performance_sport	Performance	
	“I went back to running last February.”	Restart_sport	Restart	

**Table 2:** Sociodemographic characteristics of the participants. Values with statistically significant overrepresentation (*P*<.05) are italicized.

Tool		Sport apps	MyFitnessPal	Weight Watchers	Total
Population size, n		22	20	37	79
**Gender, n (%)**					
	Men	*14 (64)*	4 (20)	1 (3)	19 (24)
	Women	8 (36)	*16 (80)*	*36 (97)*	60 (76)
**Age in years, n (%)**					
	20-29	3 (14)	*7 (35)*	1 (3)	11 (14)
	30-39	*10 (45)*	*8 (40)*	5 (13)	23 (29)
	40-49	*9 (41)*	4 (20)	11 (30)	24 (30)
	50+	0 (0)	1 (5)	*20 (54)*	21 (27)
	Total	22 (100)	20 (100)	37 (100)	79 (100)
**Occupation, n (%)**					
	Self-employed	1 (5)	0 (0)	2 (5)	3 (4)
	Upper management, experts, and professionals	*11 (50)*	4 (20)	2 (5)	17 (21)
	Intermediate professions	6 (27)	*10 (50)*	15 (41)	31 (39)
	Clerical	3 (14)	3 (15)	*15 (41)*	21 (27)
	Manual workers	0 (0)	*2 (10)*	1 (3)	3 (4)
	Unemployed	1 (5)	1 (5)	2 (5)	4 (5)
	Total	22 (100)	20 (100)	37 (100)	79 (100)

## Results

### Three Types of Users

**Figure 1:**
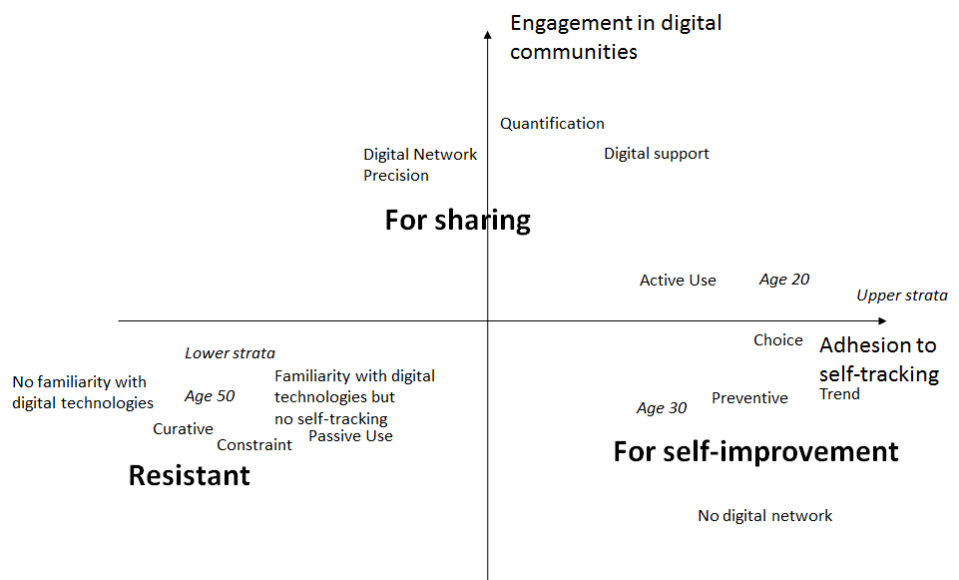
Three types of users.

Three clusters differed in their habits of self-quantification app use (Figure 1). Quadrants of Figure 1 labeled “Resistant,” “For self-improvement,” and “For sharing” indicate the position of the 3 clusters along the 2 axes (reluctance vs adhesion to self-tracking [axis 1] and rejection vs integration in the digital community [axis 2]) and the participants’ main sociodemographic characteristics. The device did not determine the use: the relationships between and within the clusters and the sample based on device used (sport app, Weight Watchers, or MyFitnessPal) represent the diversity of uses in the various classes of the typology (Multimedia Appendix 1). Between the 3 clusters, several lines of differentiation emerge (Figure 1). On axis 1 (“adhesion to self-tracking”), differences are found between the individuals most resistant to digital devices and the self-quantification process (on the left) and the individuals most connected and focused on monitoring and counting. There is an overlap between those who used self-tracking tools under constraint (on the left) and those who chose to use them (on the right). Indeed, this divide reflects the opposition between individuals who used digital devices to manage their weight gain or for a curative aim and those who used them for prevention or control of previous conditions, such as bulimia or anorexia, to thwart another trigger.

This differentiation corresponds to the opposition between poorer categories (manual and clerical workers on the left) and wealthier categories (executives on the right). Axis 2 shows the opposition between the individuals most resistant to entering a digital online community (at the bottom) and the individuals most integrated into online social media, who were also the most active contributors (at the top).

The “resistant” cluster was not only merely associated with reluctance regarding digital devices or self-quantification but also showed strong reluctance regarding online participation. The “for self-improvement” cluster combined adhesion to self-quantification, rejection of online social media, and preventive goals. The “for sharing” cluster was defined predominantly by the intense use of and active participation in a digital community that motivated, encouraged, and supported users.

### Individuals Resistant to Self-Tracking

The first cluster (“Resistant”) consisted of individuals with little inclination to use digital devices, particularly those associated with self-quantification. The majority of these individuals were women (28/32, 88%), who, with an average age of 50 years, were older than average and belonged predominantly to intermediate and clerical sociodemographic categories (Table 2).

These individuals monitored their diet (weighed food) or physical activity (notably with pedometers) and weighed themselves, but recorded little on the apps and were not familiar with the quantification processes. The majority of these individuals were Weight Watchers members living in deprived areas and former users of MyFitnessPal or physical activity trackers, who used these tools temporarily as a result of lassitude, disinterest, boredom, or meeting their goal of losing weight. For example, 1 individual was annoyed with the mobile Weight Watchers app, referring to it as “fiddle-faddle” (aged 68 years, retired), whereas someone else, who had lost weight with Weight Watchers (aged 47 years, intermediate profession) stated:

I’ll admit that since I know it by heart…I’m not really addicted to numbers and, so...well, it’s been a while since I’ve counted my points.

Among this “Resistant” group, 2 subgroups emerged. The first and larger group (23 out of 32) was composed of individuals resistant to digital apps and devices in general, with a more specific reluctance to engage in online social media, and who were unfamiliar with digital technologies: “I don’t know how,” explained Marguerite (Weight Watchers, aged 67 years, nursing assistant), although she had a tablet. However, when it came to digital devices, she cited her profession and her age as a hindrance, saying it did not allow her to familiarize herself with computers and the Internet.

These individuals seldom participated in the digital communities linked to the 3 types of apps, and, if they did, they often participated in discussion forums as spectators:

I look…just to tell myself “well, yes, we are all the same, you know.”

Weight Watchers, aged 55 years, intermediate profession

The second subgroup (9 out of 32 individuals) consisted of individuals who were resistant to the self-quantification process but familiar with digital technologies (eg, through their jobs) or in areas other than diet and fitness, such as gaming or online. Of the Weight Watchers members in this group, the nonuse of the digital app reflected their lack of motivation to follow the program. For instance, Michel (Weight Watchers, aged 58 years, self-employed) has several apps (for wine, to track his dog, and a Garmin global positioning system for his vacation itinerary), but because he was not very enthusiastic about exercise, he did not have a “fitness” app. He hardly showed any interest in trying the Weight Watchers digital program and used it inconsistently (“just the general gist”), a euphemism we interpreted to mean that he no longer really followed the program and he would soon discontinue.

### Self-Tracking Devices for Self-Improvement

The second cluster (“For self-improvement”) used self-tracking devices for self-improvement. This cluster consisted predominantly of sport app and MyFitnessPal users, who particularly appreciated the self-quantification approach because it enabled them to measure, record, and quantify themselves. This group contained more men (9/22, 41%) than the other 2 groups and, with a median age of 38 years, individuals were younger than the survey average and tended to be executives or have intermediate professions.

Individuals in this group shared the characteristics of wanting to use self-quantification tools and refusing to join a social media network: they were more concerned with personal progress than comparing their results with others. As 1 individual stated:

It’s more for myself, to improve over previous years.

Matthieu, Fitness tracker, aged 23 years, higher managerial profession

Similarly, another individual emphasized his dislike of competing with others:

Challenges don’t [do it for me], not at all. I hate that. I don’t have anything to prove except to myself. So, I’m relatively individualistic…I note my progress.

Guy, MyFitnessPal, aged 49 years, manual worker

These individuals did not compete with others, but rather measured their success by exceeding a personal goal set by themselves on the app. Some of these individuals looked for personal victories elsewhere, such as sporting competitions (9 out of the 22 individuals in this cluster); however, they still were unwilling to publish their performance data.

In addition, these individuals were motivated by congratulatory actions. Although members of the first cluster felt that the comments were intrusive or condescending (“stupid” or “silly”), members of the second cluster saw these comments as a real source of motivation:

Everyone congratulates you, comments on your activity so it makes you want to continue.

Ophélie, MyFitnessPal, aged 29 years, clerical

Finally, in this cluster, the self-tracking tool monitored the individuals’ activities, whether starting or restarting a sporting activity or preventing a wellness risk, such as weight gain. Thus, within this framework, the digital device was a very useful accessory, but only as a means of self-improvement.

### Self-Tracking for Sharing

The third cluster (“For sharing”) was composed of participants who had intense recourse to the digital community (22 out of 25), were significantly associated with active or passive engagement in the digital network, and who tended to have intermediate professions and be in their 40s. For most of these individuals, this involvement was active: they published their data, had online friends or subscriptions, were followed by other users, or regularly participated in discussion forums. Their second common characteristic was their love of quantification (significantly associated with this cluster), making them complete users of self-tracking devices (they measured, recorded, quantified, and participated in the digital community).

Twice as many individuals (12 out of 20) in this cluster than in the other clusters viewed online communities as a support system. Thus, these individuals used self-tracking tools constantly to manage their physical activities or diet on a daily basis.

In this cluster, some of the individuals looked for the most precise self-measurements possible, often reducing quantification down to the minute, second, or nearest gram (precision is significantly associated with this cluster; see Multimedia Appendix 1).

Furthermore, sporting activities or weight loss efforts were central to the lives of the individuals in this group, with many of them exhibiting strong motivation to achieve their goals. The largest weight loss “successes” (up to 45 kilos) were found in this cluster. So far, it is difficult to assess the sense of the causality: the use of digital device led to success or the most motivated interviewees took advantage of all their device’s functionalities. Digital devices and self-quantification tools were ends in themselves.

### Diversity of Uses and Motivations

The utilization of digital devices differed drastically from one individual to another, whether in regard to the functionalities used, data taken into account, or integration in a digital community. Between individuals, measuring, recording, quantifying, and communicating were done very differently.

### Converting Measurements to Written Records

The reasons why individuals measured dietary intake or physical activity were diverse. Among the Weight Watchers members, weight loss was the ultimate goal for esthetic or health reasons. Users of MyFitnessPal had more varied goals; in most cases, although, the aim was a slimmer physique. With this aim, the app was either used for a long period of time (several months) or for a short period of time, ranging from 2 to 3 weeks, possibly several times a year. Some individuals used the devices to gain muscle, whereas others used them to monitor a medical condition, such as diabetes or an eating disorder (anorexia or bulimia), using the app as a safeguard to ensure they neither ate too much nor too little. Sport app users used the digital devices either to accompany the start or restart of sporting activities or to improve their performance at multiple levels, particularly in individual sports, such as running, cycling, and swimming.

Analysis showed that users had diverse reasons for recording their food intake or physical activity. Counting, recording, and writing are central tools in the Weight Watchers program [22]. The awareness afforded by converting measurements into a written record was evoked more broadly by the users of the 3 apps studied. Taking notes was also an obligation to control oneself (“a little police officer in your pocket,” said Clémence, MyFitnessPal, aged 41 years, intermediate profession).

MyFitnessPal is based on keeping a food diary. Differences between uses were found, eg, in the recording of excess. Some users published their excesses as a way to actualize and recognize them; however, others preferred to keep their excesses private. Differences were also found in the measurements scrutinized by the users, with some users reviewing overall calorie intake, and others attentively examining all nutritional data, such as calories, protein, carbohydrates, sodium, potassium, cholesterol, vitamins, and calcium.

Recording all food intakes was not seen as a time-consuming or fastidious constraint. Information was recorded after each meal, at the end of the day, or even at the start of the day when meals were being planned or during a spare minute or designated moment. For many, this recording session was seen as a way to make time for oneself outside of domestic or professional obligations. During this process, the speed and automation offered by digital support were widely viewed as an advantage, whether in calculating points or calories or in populating the database.

Use of sport apps varied widely and was strongly linked to the intensity of the users’ sporting activities. Some individuals, mostly the least athletic, used the tool daily to measure and record all of their physical activity (total number of steps, going up the stairs at home, etc), showing an interest in accumulating data on all movements made. Other, more athletic individuals, only measured what they considered to be true physical activity (an activity for which a specific time was allocated and for which real physical effort was implicated). The data used were also variable: distance, speed, incline, improvement from one session to the next, and heart rate are available. Some users, mostly the most active, would consult all current and past data, analyzing all parameters with precision. Conversely, other users were content with the basic use of the device to measure average speed and distance covered.

### Quantification: The Power of Numbers

Quantification capabilities offer several key benefits to individuals. First, numerical measurement provides objectivation: for instance, Christian was able to monitor and “match objective data to a personal feeling” (sport app, aged 49 years, intermediate profession). Participants greatly appreciated presentation in graphical format because it was easier to read and detect objective trends. Representing activities using mathematical expressions, such as curves, graphs, diagrams, or evolutions over time, also lent a scientific appearance to a mundane activity, such as walking, running, or weighing oneself, and thus appeared more valuable.

Second, numbers are authoritative. One user, Laurent, explained:

The numbers are there…They are certain.

sport app, aged 31 years, higher managerial profession

This certainty was just as important to him as his weight gain, as ceasing participation in sports to start an intense job caused him to lose confidence in himself despite his excellent professional profile. For some, measuring even went as far as giving the activity its value—its very existence. As stated by one user who would occasionally forget his cardio belt or leave his watch at home:

When I do, I’m working in the dark. I mean, without information.

sport app, aged 44 years, higher managerial profession

In these cases, an activity not measured by the device did not count, as if it never happened, although it could have been recorded manually and thus counted.

Quantification is a way to manage or prevent a condition. For example, one user wanted to “get ahead” of age-related weight gain:

At some point, you see the years piling up and start to think: it’s time to get your act together…After all, I’m a little scared of…I’m trying to make sure I don’t put on that one invisible kilo every year for 10 years when I’ll realize there are all those 10 kilos.

Benoit sport app, aged 38 years, higher managerial profession

Another user expressed the need to start exercising after a heart attack:

I need to exercise for my health, but exercise is annoying. That’s just the way it is. When you’re a kid, you exercise for fun, and when you’re old you exercise because you need to take care of yourself.

Emile, sport app, aged 44 years, higher managerial profession

Finally, the quantification process applied to 2 types of profiles that differed along a trend versus precision axis (see quotes in Table 1). The first profile consisted of individuals who simply wanted to see trends, whereas the second profile consisted of individuals who wanted numerical precision.

### Sharing: Engagement in Social Networks

In the digital domain, there is a high diversity of engagement levels: the majority of users are passive readers, some are spectators, a small number are occasional participants who become involved based on their interests, and an even smaller number are active contributors [23]. Engagement paths were taken: at first, an individual read the forum content, looked at other users’ performances, observed, and then started posting after a familiarization process that leads to self-exposure by publishing one’s own words or data.

The online community was a major source of motivation for 3 different reasons. First, the community provided support: discussions with friends or even the encouraging messages from the app provided motivation because individuals with the same concerns, interests (sports), or struggles (excess weight) were brought together. As stated by one user, the community was seen as “benevolent”:

I was really feeling down, and it lifted me up…The community is extremely caring.

Elisabeth, Weight Watchers, aged 45 years, intermediate profession

The community was based on a collective identity. Therefore, discussions with others proved particularly useful when users faced difficulties, such as giving into temptation or hitting a weight-loss plateau.

The community also provided positive identification models and access to shared experiences:

Going to a community and meeting people who are in the same situation as you or who started MyFitnessPal two years ago and have lost 35 kilos, those are the people you want to follow; it’s their advice you want to have.

Valentine, MyFitnessPal, aged 29 years, higher managerial profession

The digital community was also a source of knowledge. The transformation of an individual into an expert on his or her own health is triggered by recourse to the Internet with a health perspective and leads to an increasing digital divide [24]. Individuals in the upper and middle groups received or gave advice, read discussions, and, after a personal information selection process, assimilated their own knowledge. Ophélie (MyFitnessPal, aged 27 years, intermediate profession), who lost 20 kg, found a lot of information on the Internet, but the community gave her access to opinions. Experience was what she trusted:

And after, it was thanks to the forum, where everyone shares their meal plans, their ratios, so I experimented a lot until I found what I liked…

Finally, and specifically for the sport apps users, the community was a source of emulation because it formed a pool of rivals who fueled some users’ taste for competition, a rather masculine attitude [25]. Competing with others—known or strangers—sharpens practice: the social network offers a challenge. Sport apps’ users published their results to advertise their performances, gaining a sense of satisfaction and perseverance by beating others and in proving their skills through sports data sharing sites, such as Strava. As stated by one participant, with Strava:

You know what you have to beat [the record of the guy on the segment] so it's pretty fun.

sport app, aged 42 years, self-employed

Thus, the question is raised as to what extent these various uses can be explained by social factors.

## Discussion

### Digital Inequalities

With the increase in digital technologies and health inequalities, the notion of the digital divide in relation to social status [26] must be examined (Textbox 1).

The use of self-tracking tools was socially divided: the individuals most adept at self-quantification were also those who belonged to the more affluent milieus. This divide corresponds to axis 1 of the factor analysis, which corresponds to the central dichotomy between high-income socioeconomic categories and poorer categories.

This study expands research on digital inequalities. Belonging to an affluent social milieu intrinsically involves elements that encourage self-tracking (Textbox 1): owning efficient tools that limit technological hindrances (slow connection and session interruptions, with some studies showing that the interaction speed of apps has a significant effect on user satisfaction [27]), familiarity (through work or education) with the Internet and new technologies, concern for recommended healthy lifestyles [28], and tracking food and weight from a perspective of prevention, with the connection of health to daily diet being viewed as a long-term relationship [29].

Although self-tracking is not a practice reserved for elite members of society, it is often seen as one, and it is appreciated by members of intermediate professions; by imitating members of the elite, the middle class perceives the use of self-tracking as a way to access the practices of the next highest social group to which they aspire. “Personalized” self-tracking tools allow them to avoid, particularly when they are overweight, the gaze of those who dominate them socially or of medical bodies supervising them.

Conversely, in poorer milieus, some individuals evoked their lack of technical skills, a hindrance frequently mentioned to explain why they did not use digital devices.

Blank and Reisdorf [30] have explained active and passive attitudes to publishing on the Web: the 2 most decisive variables are the ease of publishing data on the Internet versus uncertainty in one’s ability to publish one’s own data on the Internet. Our results suggest that this uncertainty can be interpreted as a feeling of cultural illegitimacy [31] about using digital devices among members of poorer milieus, who may feel that using ICT oversteps their social position.

### Dynamic Dimensions: Life-Course Transitions and Turning Points

Dynamic dimensions linked to life course provided additional insight (Textbox 1). The youngest individuals (aged 18-24 years) and those in the highest social category are most likely to have mobile phones and most frequently use an Internet connection. Hence, the most resistant to digital devices in our survey were also the oldest interviewees, and these findings confirmed previous observations [32].

However, the average age of users for whom digital devices were most central was 40 years. Thus, our study showed that recourse to self-tracking tools was more linked to a specific position in life course, a factor which exercises a fundamental influence on food choices [33] and body governance, now in the area of new technologies. Some individuals turned to digital devices after a turning point in their life course (new job, new home, or divorce), which led to major changes in dietary and sporting practices. Quantifying and tracking data were therefore ways to bring order back to a life that had been temporarily disrupted [2].

For other individuals, the use of digital devices was prompted by a “midlife” transition, which implied small adjustments in food choices or physical activity to prevent weight gain. In these cases, self-tracking tools were used, either regularly or constantly, by individuals who were approximately aged 40 years.

These concerns were more distant for younger individuals in their 20s, who viewed the bodily horizon in much more serene terms. Their use of smart watches was more irregular, and, in their opinion, less necessary: the pleasure they found in physical activity dominated.

Finally, the perception of a health risk that was directly linked to a family member’s illness or a life course turning point could incentivize interviewees to monitor their diets or physical activity.

**Social factors in the use of self-tracking tools.**

**Hierarchical factors**

- Efficient equipment
- Digital familiarity and cultural legitimacy
- Diet, a health factor
- Preventive aim

**Dynamic factors**

- Age
- Midlife transition
- Life-course turning points

**Heterogeneity factors**

- Social integration and cultural intermediaries
- Anteriority of written record-keeping
- Awareness of a risk

### Cross-Cutting Factors of Heterogeneity

Cross-cutting factors moderated the ascription of social status (Textbox 1). The degree of social integration was a major factor that either promoted or hindered the use of digital apps. Indeed, good social integration facilitated the spread of an innovation by imitation. Our results extended previous research: good social integration also fosters the adoption of new ideas and objects because of the intervention of the opinion leader [34], who is close to individuals from a social standpoint, but still retains a slightly higher position. As cultural intermediaries, they are seen as experts and further the spread of new practices linked to digital technologies.

Regardless of social milieu, the use of digital self-quantification tools was associated in part with previous acts of keeping written records or quantification. Of the people surveyed in this study, one-fourth mentioned previous habits of keeping a written record of physical activity or a food diary.

### A Divide in Terms of Uses

Although we corroborated previous studies [24] and found a digital divide, it was less associated with equipment and more with type of use. Indeed, the emergence of digital diet and fitness tools coincided with the preoccupation of members of intermediate and higher sociodemographic categories, who had similar characteristics in terms of food, health care, and body care, with controlling their diets and physical activity. Self-tracking tools gave them a new, faster, more precise, and often more enjoyable way to monitor and control their diets and physical activity. Conversely, in poorer milieus, neither health through diet nor weight control nor physical exercise were priorities.

For all these reasons, self-tracking tools definitely increased motivation for people who wanted to lose weight or be more physically active. However, although individuals who lost a lot of weight or were very physically active used digital apps, sometimes intensely, it cannot be concluded that the use of these tools was the reason for their success. Rather, using a self-tracking tool was a reflection of the motivation to control one’s weight or exercise.

### Limitations

The survey was predominantly conducted among current users of digital devices, who were willing to share their experiences because they were satisfied with the outcomes. Further research is needed among former users and intermittent users, and among those who are reluctant to implement digital technologies and self-quantification practices in all social milieus, to augment the research presented here.

### Strengths

A particular strength of our research is that it is one of the few qualitative studies on digital apps based on such a large sample of in-depth interviews [35]. Another strength of our study was the reliability of our data (number of interviews, ethnographic observations, quantitative analysis, and triangulation of methods).

### Conclusions

The results of this study have major implications for public health. Two major divides were highlighted, with significant social implications. Those most willing to use self-tracking tools belonged to affluent milieus, for whom self-care of the body was an ethic, and intermediate milieus, where the cultural desire to “eat better” or “move more” was made practical thanks to digital devices. In both cases, a preventive outlook when it came to healthy lifestyles was a motivator to use self-quantification devices. Among low-income milieus, there was more frequent reluctance, either to digital devices in general or to the self-tracking process. Moreover, members of poorer milieus showed more marked reluctance to speak out in digital communities, whereas members of the middle class found motivation, support, and an arena for expression in digital communities. Our final major finding was that it was neither intrinsically the digital device nor the app that motivated individuals to modify their diet or physical activity toward improved health behaviors: it was rather the active way in which engaged individuals used the devices and apps.

## Appendix

Multimedia Appendix 1 Supplementary tables and legends.

## Abbreviations

ICT: information and communication technology
WW: Weight Watchers
