# Supplementary material for: Digital Inequalities in the Use of Self-Tracking Diet and Fitness Apps: Interview Study on the Influence of Social, Economic, and Cultural Factors
Source: JMIR Mhealth Uhealth. 2018 Apr 20;6(4):e101. doi: 10.2196/mhealth.9189 (PMC5935808; doi:10.2196/mhealth.9189)
Supplement: Multimedia Appendix 1 [file mhealth_v6i4e101_app1.pdf]

Supplementary File

Acknowledgments

The authors like to thank the INRA's Did'It Metaprogramme Board for the financial support, and the NutriPerso coordinator, Louis-Georges Soler, for his support, Marie Plessz, Sylvie Fainzang and Anne-Sylvie Pharabod for stimulating discussions, the management of Weight Watchers® France and the leaders of the meetings that we attended for their especially warm welcome, and the participants who agreed to be interviewed.

Softwares used

For content analysis of the interview data, we used Hyperbase software (CNRS – Université Nice Sophia Antipolis, Nice, France). Hyperbase, which can be used for any language with a roman alphabet, offers several functionalities, such as word counts or measurements of textual specificities of interviews.

For the quantitative analysis, we used Stata®.

Figure 1. PCA: Correlation circle axes 1 and 2 of the 25 practices

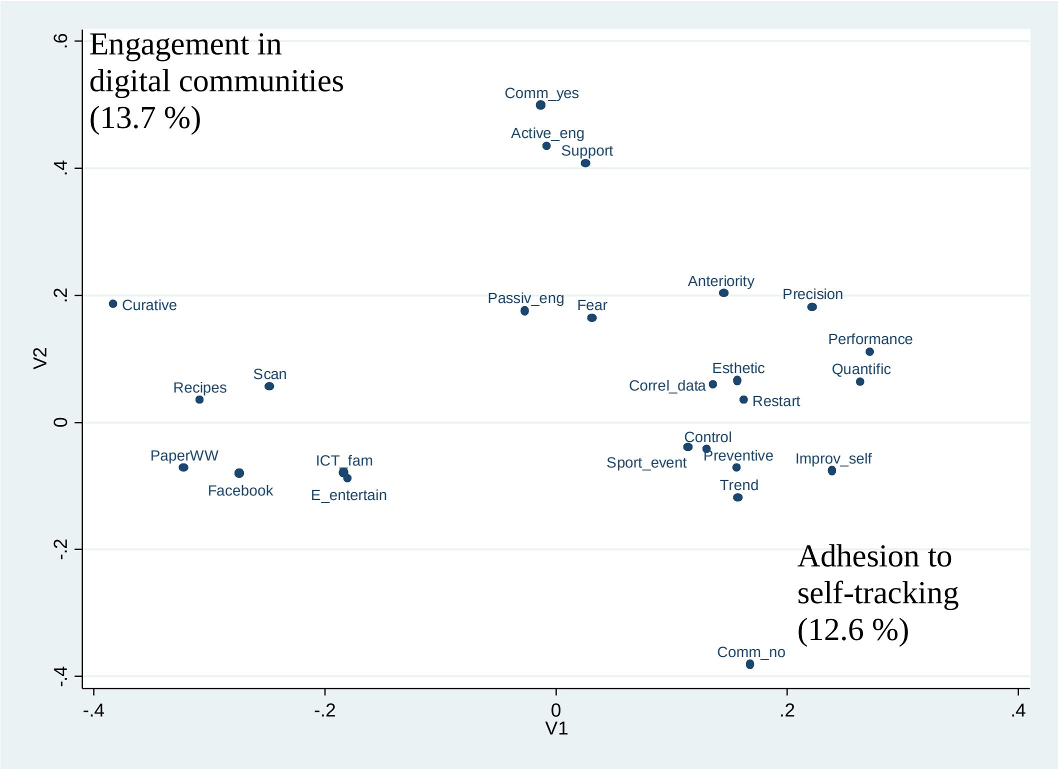

Footnote: Axis 1 is interpreted as "adhesion to self-tracking", and axis 2 as "engagement in digital communities". Percentage of variance is between parentheses. See abbreviations in Table 1 below.

<u>Figure 2. PCA: Principal plan axes 1 and 2 of descriptive sociodemographic variables</u>

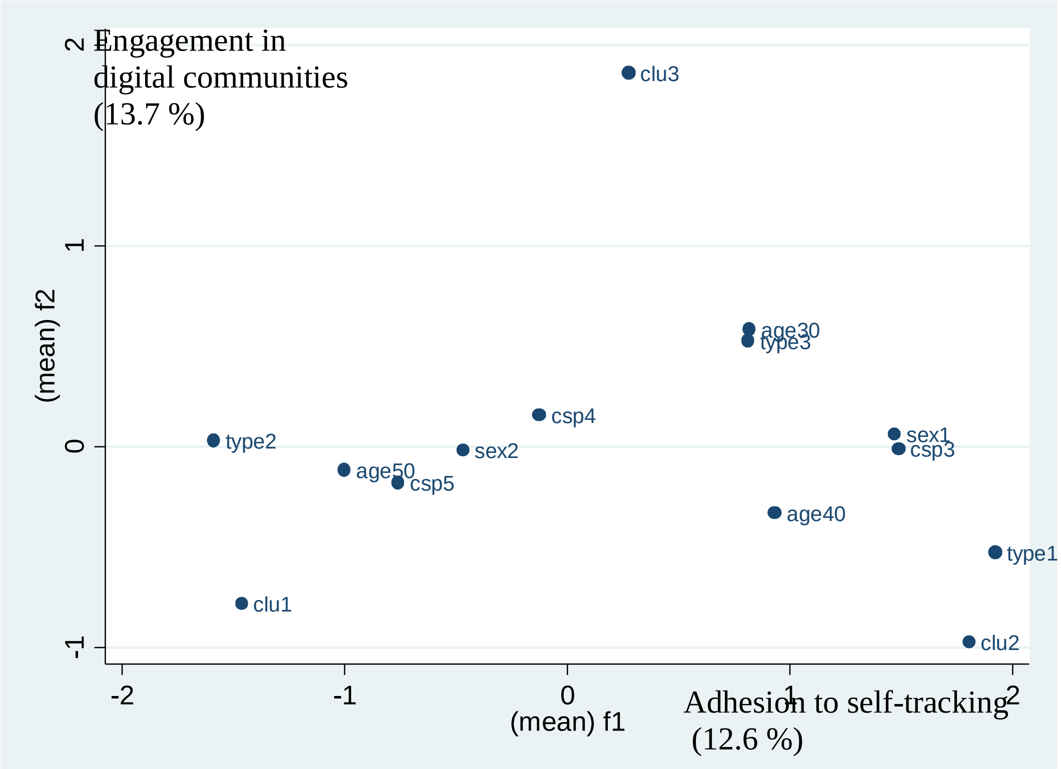

Footnote: Axis 1 is interpreted as “adhesion to self-tracking”, and axis 2 as “engagement in digital communities”. Percentage of variance is between parentheses. See abbreviations in table 1 below.

Table 1 – Abbreviations of descriptive and socio-demographic variables

**Variable**	**Abbreviation**
Male	sex1
Female	sex2
Aged 20 to 29	age 20
Aged 30 to 39	age30
Aged 40 to 49	age40
Aged 50 and more	age50
Upper management, experts, and professionals	csp3
Intermediate professions and self-employed	csp4
Clerical and routine white and blue collar workers	csp5
Cluster 1	clu1
Cluster 2	clu2

Cluster 3	clu3
Sport apps users	type1
Weight Watchers® members	type2
MyFitnessPal users	type3

Table 2 - Correlation between active variables and cluster (Phi coefficient)

**Themes**	**Active variables**	**Cluster 1**	**Cluster 2**	**Cluster 3**
Functionalities	Quantific	-0.3895*	0.1568	**0.2600***
	Scan	0.1456	-0.2460*	0.0834
	Recipes	**0.2905***	-0.2968*	-0.0206
	Facebook_WW	**0.3063***	-0.1516	-0.1772
Uses	Improv_self	-0.2271*	**0.4097***	-0.1552
	Challenge	-0.0729	0.1141	-0.0330
	Precision	-0.3337*	0.1312	**0.2258***
	Trend	-0.2145	**0.4184***	-0.1769
	Correl_data	-0.2573*	**0.3031***	-0.0206
Digital network	CommuYes	-0.4144*	-0.2974*	**0.7240***
	CommuNo	0.0639	**0.5436***	-0.5914*
	Passiv_eng	-0.3141*	-0.1516	**0.4776***
	Active_eng	-0.2233*	-0.1726	**0.4020***
	Support	-0.2959*	-0.1339	**0.4413***
Relation to publishing	Anteriority	-0.2432*	0.0280	**0.2298***
	PaperWW	**0.5379***	-0.2757*	-0.3020*
	Fear	0.0554	-0.1781	0.1131
Familiarity with ICT	ICT_fam	0.1922	-0.0128	-0.1906
	E_entertain	**0.3150***	-0.1615	-0.1769
Reasons for use	Esthetic	-0.2774*	**0.2878***	0.0154
	Preventive	-0.2145	**0.4184***	-0.1769
	Illness_management	-0.2145	-0.0455	**0.2702***
	Curative	**0.4315***	-0.5049*	0.0312
	Performance_sport	-0.3618*	**0.2228***	0.1672
	Restart	-0.2770*	**0.4467***	-0.1382

Table 3. Cluster / population sample ratio

	Cluster			Total
**Sample**	1	2	3	Total
Sports	9.09	68.18	22.73	100.00
Weight Watchers	64.86	5.41	29.73	100.00
MyFitnessPal	30.00	25.00	45.00	100.00
Total	40.51	27.85	31.65	100.00

Table 4. Listing of the PCA: axes and eigenvalues

Principal components/correlation   Number of obs = 79
Number of comp. = 25
Trace = 25
Rotation: (unrotated = principal)   Rho = 1.0000

Component	Eigenvalue	Difference	Proportion	Cumulative
Comp1	3.42315	.275815	0.1369	0.1369
Comp2	3.14734	1.22276	0.1259	0.2628
Comp3	1.92457	.134481	0.0770	0.3398
Comp4	1.79009	.056853	0.0716	0.4114
Comp5	1.73324	.207756	0.0693	0.4807
Comp6	1.52548	.122592	0.0610	0.5418
Comp7	1.40289	.233915	0.0561	0.5979
(…)				

Principal components (eigenvectors)

Variable	Comp1	Comp2	Comp3	Comp4	Comp5	Comp6	Comp7
Quantific	0.2638	0.0634	-0.0830	0.0723	0.5046	-0.1042	0.1992
CommuYes	-0.0124	0.4988	-0.0989	0.1562	-0.0198	-0.1058	-0.0770
CommuNo	0.1686	-0.3817	0.1522	-0.0225	0.3691	0.1318	0.0533
Passiv_eng	-0.0263	0.1751	-0.4682	0.2844	0.0336	0.0083	0.1635
Active_eng	-0.0078	0.4348	0.3113	-0.0374	-0.0111	-0.0667	-0.2328
Support	0.0264	0.4075	0.3432	-0.0751	0.0355	0.1044	-0.0309
Scan	-0.2475	0.0556	-0.0725	0.0662	0.2823	-0.0010	-0.0848
Recipes	-0.3081	0.0348	0.0883	0.0533	0.1797	-0.2085	0.1155
Anteriorit~	0.1460	0.2029	-0.2401	0.1897	0.0330	0.1402	0.1024
Fear	0.0317	0.1641	0.2759	-0.1717	0.0170	-0.0657	0.4656
ICT_fam	-0.1835	-0.0802	0.0962	0.2739	0.0726	0.1713	0.1542
Facebook	-0.2734	-0.0813	0.1485	0.1901	0.2414	0.0117	0.0114
Improv_self	0.2398	-0.0770	0.2060	0.1637	-0.0933	-0.3103	0.0866
Sport_event	0.1151	-0.0392	0.0168	-0.1387	-0.1390	-0.1829	0.4922
PaperWW	-0.3217	-0.0719	0.0525	-0.1022	-0.1987	-0.0781	-0.2701
E_entertain	-0.1798	-0.0891	0.1063	-0.1501	-0.3964	-0.0026	0.3301
Precision	0.2221	0.1812	-0.1359	0.0262	-0.0722	0.0368	0.1371
Trend	0.1578	-0.1189	0.2423	0.2281	0.0780	-0.2324	-0.1885
Correl_data	0.1366	0.0591	0.0321	-0.3502	0.1947	0.4564	-0.0408
Esthetic	0.1575	0.0653	0.0773	-0.0944	-0.1049	0.4954	0.0112
Preventive	0.1572	-0.0718	0.2464	0.3431	-0.1294	0.2434	-0.1070
Control	0.1311	-0.0427	-0.2640	0.0474	-0.2540	-0.0117	-0.1218
Curative	-0.3827	0.1861	0.0708	0.0307	0.1342	0.1152	0.2171

```
 Performance |   0.2724    0.1107    0.0350   -0.2935    0.1232   -0.3515   -0.1646
     Restart |   0.1632    0.0349    0.2655    0.4773   -0.1732    0.0842    0.0978
-------------------------------------------------------------------------------------
```
